# *STK11* rs2075604 Polymorphism Is Associated with Metformin Efficacy in Chinese Type 2 Diabetes Mellitus

**DOI:** 10.1155/2017/3402808

**Published:** 2017-07-09

**Authors:** Qingchu Li, Cuilin Li, Haoyun Li, Liu Zeng, Zhiqiang Kang, Yu Mao, Xinyue Tang, Panpan Zheng, Li He, Fang Luo, Zhi Li

**Affiliations:** ^1^Department of Clinical Pharmacology, Xiangya Hospital, Central South University, Changsha 410008, China; ^2^Institute of Clinical Pharmacology, Hunan Key Laboratory of Pharmacogenetics, Central South University, Changsha 410078, China; ^3^Department of Medical Center Transformation, Zhengzhou Central Hospital, Zhengzhou University, Zhengzhou 450007, China; ^4^Department of Key Laboratory of Endocrinology, Zhengzhou Central Hospital, Zhengzhou University, Zhengzhou 450007, China

## Abstract

Metformin is a classical oral antidiabetic drug, often recommended to be the first-choice treatment of type 2 diabetes mellitus (T2DM). Based on the previous research on *STK11* and diabetes, we aimed to investigate the distributive characteristic of *STK11* rs2075604 polymorphism and the potential influence of *STK11* rs2075604 polymorphism on metformin efficacy among Chinese T2DM patients. There was no significant difference between T2DM patients (*G* = 64.8%, *T* = 35.2%) and healthy subjects (*G* = 62.7%, *T* = 37.2%) in *STK11* rs2075604 genotype and allele frequencies. After 12 weeks of treatment, 62 patients were defined as the responders and 32 patients as nonresponders according to the decrease of HbA1c level. And the GT + TT genotype in *STK11* rs2075604 can decrease HbA1c level more significantly than the GG genotype. Furthermore, the allele frequency of T in the *STK11* rs2075604 was higher in the responders than the nonresponders (43.55% versus 26.56%). The T allele in the *STK11* rs2075604 had a 2.133 times great chance of responding to metformin treatment. In conclusion, this study suggested that the *STK11* rs2075604 genetic polymorphism was significantly associated with metformin efficacy in Chinese T2DM patients and the carriers of the T allele may gain a better therapeutic metformin efficacy compared with the G allele. This trial is registered with clinical study registration number NCT03155087.

## 1. Introduction

As we all know, metformin is a classical drug used for treating type 2 diabetes mellitus (T2DM) in more than 50 years and is recommended to be the first-choice treatment of T2DM in the recent international guidelines [1]. The pharmacological mechanism of metformin is to increase the utilization efficiency of plasma glucose by enhancing a body's sensibility of insulin. Adenosine monophosphate protein kinase (AMPK) is an important regulator of glucose and lipid metabolism, which plays a vital role for metformin efficacy [2, 3].

However, more and more published knowledge reported that some of T2DM patient cannot respond adequately to metformin, and there are some individual differences in using metformin to treat T2DM [4]. On the other hand, about 10–20% of patients are not able to tolerate the side effects of metformin [5]. According to the former research, some genetic polymorphisms in genes encoding proteins, such as *SLC22A1* [6, 7], *OCT1* [8], and *IL-1B* [9], are responsible for the pharmacokinetics or pharmacodynamics of metformin. Recently, the AMPK pathway and its regulators are gaining the attention for the difference of the metformin effect.

Serine-threonine kinase 11 (STK11), also known as LBK1, is an important upstream activator of AMPK which can phosphorylate within the activation loop of the *α*-subunit [10]. Legro et al. [11] demonstrated that the genetic polymorphism of *STK11* was associated with the effect of metformin in treating polycystic ovarian syndrome. Bassols et al. [12] found that the G allele for rs8111699 in *STK11* was a protective factor for gestational diabetes mellitus. Shaw et al. [13] reported that the kinase *LKB1* (*STK11*) would mediate the therapeutic effect of metformin in lowering blood glucose.

Therefore, this study aimed to investigate the demographic and clinical characteristics between T2DM patients and healthy subjects and evaluate the prevalence of the genotype and allele frequencies of *STK11* rs2075604 polymorphism in T2DM patients and healthy subjects. Further, this study hypothesized that the genetic polymorphism of *STK11* will influence the metformin efficacy in treating Chinese T2DM patience.

## 2. Material and Methods

### 2.1. Subjects

This study was conducted in 400 T2DM patients (245 males and 155 females) and 200 healthy subjects (111 males and 89 females). All subjects were recruited from Zhengzhou Central Hospital during the period of July 2013 to December 2015. The criteria for enrollment were as follows: diagnosed with T2DM in keeping with the WHO criteria in 1999 [14], with fasting blood glucose (FBG) ≥ 7.0 mmol/L and/or postprandial blood glucose (PBG) ≥ 11.1 mmol/L and not using other antidiabetic drugs within the experimental period. Patients diagnosed with other significant illnesses such as cancer, myocardial infarction, stroke, severe liver, and kidney disease were excluded. This study was approved by the Ethics Committee of Zhengzhou Central Hospital, Zhengzhou University (Zhengzhou, Henan, China; registration number 2016ys5), and all the subjects had written informed consent before participating in the study.

A series of clinical information including metformin dose, glycosylated haemoglobin (HbA1c), fasting blood glucose (FBG), postprandial blood glucose (PBG), family history, complications, and body mass index (BMI) was collected from each patient at the baseline and ending point. However, due to disease progression, 306 patients were treated with other antidiabetic drugs within the experimental period. Therefore, 94 T2DM patients treated with metformin were enrolled in a final efficacy analysis only. Metformin dosages ranged from 500 to 2000 mg per day among individuals and were adjusted according to the changes of glucose. Based on the response to metformin, these T2DM patients were divided into two groups, the response group (decrease in HbA1c levels was by more than 0.5% from the baseline) and nonresponse group (decrease in HbA1c levels was less than 0.5% from the baseline) [7, 8].

### 2.2. DNA Isolation and Genotyping

Three milliliters of venous blood were collected from each subject using an EDTA anticoagulant tube. Genomic DNA was extracted from peripheral blood leukocytes by a Wizard Genomic DNA Purification Kit (Promega, Madison, USA) according to the manufacturer's protocol, and the samples were stored at −20°C. High-throughput MALDI-TOF technology was applied to genotyping. The primers of *STK11* rs2075604 used for polymerase chain reaction (PCR) were ACGTTGGATGAAGGAGACGGGAAGAGGAG (forward) and ACGTTGGATGATATATCCTTTCCGGTGTT (reverse). After that, the PCR products were sequenced by a MassARRAY Analyzer 4.0 (Sequenom Inc.).

### 2.3. Statistics

Data was analyzed using SPSS 18.0. (Chicago, Illinois, USA). The Pearson chi-square (*χ*^2^) test was used to analyze the Hardy-Weinberg Equilibrium (HWE) and allelic frequencies in different groups. The demographic and clinical characteristics obeying the normal distribution in T2DM patients and healthy subjects were compared by the two-sample *t*-test, while the skewed parameters by the Mann–Whitney *U* test. The two-sample *t*-test was also used to estimate the follow-up data obeying the normal distribution and Wilcoxon's signed rank test for nonparametric data. What is more, we evaluated the association between metformin efficacy and alleles using logistic regression by adjusting for sex, age, and BMI. In our statistical analysis, two-tailed *P* values less than 0.05 were considered as significant.

## 3. Results

### 3.1. Clinical Characteristics of Subjects

For a genotype and allele frequency analysis, a total of 400 T2DM patients and 200 healthy subjects were enrolled. The demographic and clinical characteristics of T2DM patients and healthy subjects are shown in Table 1. The BMI (24.46 ± 2.28 kg/m^2^ versus 22.68 ± 1.69 kg/m^2^), HbA1c (7.75 ± 1.75% versus5.53 ± 2.84%), FBG (7.82 ± 2.53 mmol/L versus 5.37 ± 1.55 mmol/L), PBG (15.22 ± 6.43 mmol/L versus 6.56 ± 2.97 mmol/L), and insulin (INS) (7.99 uIU/mL versus 5.80 uIU/mL) of T2DM patients were significantly higher than those of the healthy subjects, while C-peptide (CPO) (1.89 ng/mL versus 2.35 ng/mL), postprandial C-peptide (PCPO) (5.45 ± 3.44 ng/mL versus 7.48 ± 4.01 ng/mL), and high-density lipoprotein cholesterol (HDL) (1.09 mmol/L versus 1.40 mmol/L) of T2DM patients were significantly lower than those of the healthy subjects. Moreover, there were no significant differences in total cholesterol (TC), triglycerides (TG), and low-density lipoprotein cholesterol (LDL) between these two groups.

### 3.2. Genotype and Allele Frequency Distribution

The genotype and allele frequencies of *STK11* rs2075604 polymorphism in T2DM patients and healthy subjects are shown in Table 2. Both patient and healthy groups were in agreement with the Hardy-Weinberg equilibrium (*P* value nonsignificant, Table 2). The minor allele frequencies (MAFs) of *STK11* rs2075604 in T2DM patients and healthy subjects were 35.2% and 37.2%, respectively. No significant differences were presented in genotype frequency (*P* = 0.355) and allele frequency (*P* = 0.488) between T2DM patients and healthy subjects, indicating that *STK11* rs2075604 polymorphism has no remarkable impact on the incidence of T2DM in the Han Chinese population.

### 3.3. Comparison of Clinical Characteristics between the Responders and Nonresponders before and after Metformin Treatment

94 T2DM patients treated with metformin for consecutively 12 weeks were analyzed for the therapeutic efficacy of metformin (Table 3). Among these enrolled patients, 62 (34 males and 28 females) were defined as responders and 32 (20 males and 12 females) as nonresponders according to the grouping standard. The adjusted mean metformin dosages of the response group and nonresponse group were 1463.71 mg/d and 1412.50 mg/d, respectively (*P* value nonsignificant). Compared with the nonresponse group, the mean change of HbA1c (−2.81 ± 1.57% versus −0.28 ± 0.74%, *P* < 0.001), FBG (−2.83 ± 1.94 mmol/L versus −0.83 ± 2.69 mmol/L, *P* < 0.001), and PBG (−6.76 ± 10.98 mmol/L versus −2.07 ± 5.11 mmol/L, *P* < 0.05) were significantly higher in the response group. However, we found no significant mean change in BMI, and there were no differences in age and gender between these two groups.

### 3.4. Analysis of *STK11* rs2075604 Polymorphism on Metformin Efficacy in T2DM Patients

After treatment of metformin consecutively for 12 weeks, some differences in genotype and allele frequencies in terms of *STK11* rs2075604 genetic polymorphism appeared between the responders and nonresponders (Table 4). When the comparison was made between the wild type (GG carrier) and GT carrier, the genotype of *STK11* rs2075604 polymorphism was a significant influence for metformin efficacy adjusted for age, sex, and BMI (*P* = 0.019). Compared with that of the wild type, the OR value of the GT carrier was 3.091, which meant that the therapeutic efficacy of metformin was better in the GT carrier of *STK11* rs2075604 than in the wild type T2DM patient. However, no significant difference was found between the TT carrier and the wild type regarding to metformin efficacy. For allele frequency, the result was similar with the genotype analysis that better metformin efficacy was found in the T allele. In the dominant model, when the *STK11* rs2075604 GG genotype was made to be the reference, the GT/TT genotype was linked to a significantly better therapeutic efficacy of metformin (GG versus GT/TT: adjusted OR = 3.143, 95% CI = 1.293–7.638, *P* = 0.011). In the recessive model, however, there was no difference between the GG/GT genotype and TT genotype.

## 4. Discussion

Diabetes is the most common chronic disease around the world; despite the advanced treatment, there is still heavy public health burden for more and more T2DM patients [15]. Among the varieties of oral hypoglycemic drugs, metformin is the first choice for treating the initial T2DM patients worldwide including China [16, 17]. In our study, we found that *STK11* rs2075604 genetic polymorphism had a significant association with metformin efficacy in T2DM. Patients with the GT carrier could achieve a better therapeutic effect than those with the GG carrier (the wild type). And the GT carrier in the *STK11* rs2075604 had 3.091 times of response to metformin treatment compared with the GG carrier. As far as we know, this is the first study that investigates the relationship between *STK11* rs2075604 genetic polymorphism and metformin therapeutic efficacy in Chinese T2DM patients.

**Figure 1:**
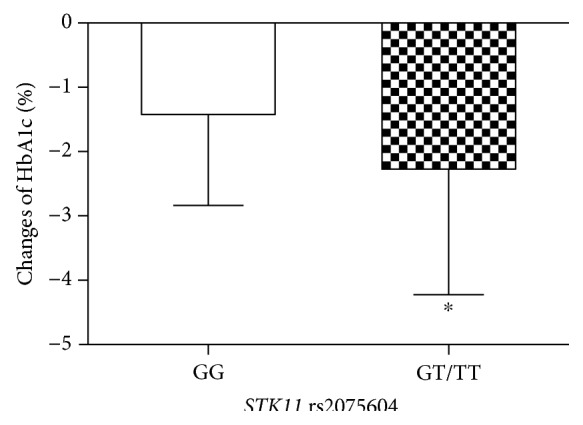
The decreased level of HbA1c in T2DM patients with different *STK11* rs2075604 genotypes after metformin treatment. Data are shown as mean ± SD. ^∗^*P* < 0.05 compared with the GG genotype group (*n* = 94).

**Figure 2:**
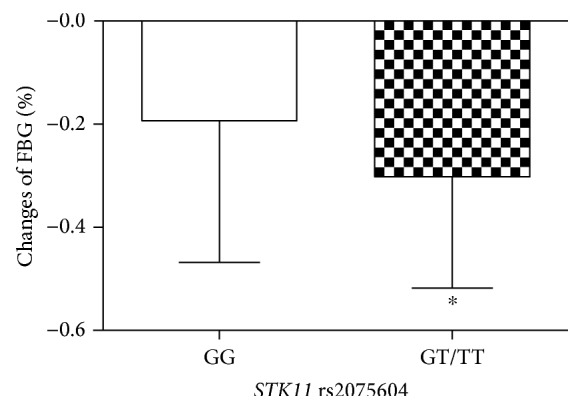
The decreased level of FBG in T2DM patients with different *STK11* rs2075604 genotypes after metformin treatment. Data are shown as mean ± SD. ^∗^*P* < 0.05 compared with the GG genotype group (*n* = 94).

AMPK is an important target of metformin that regulates the function of pancreatic *β*-cell and the secretion of insulin [18, 19]. AMPK and STK11 are related to the susceptibility of T2DM, gestational diabetes, muscle disease, and cancer [12, 20–22]. And STK11 has been reported to regulate blood glucose levels of metformin [13]. In Spanish hyperinsulinemic girls, the *STK11* rs8111699 was demonstrated to influence insulin sensitivity and metformin efficacy. The C allele was reported to be associated with lower insulin and IGF-1 levels [23]. Thus, we investigated the association of *STK11* genetic polymorphism and metformin efficacy of T2DM in this study. And we found that the *STK11* rs2075604 genetic polymorphism was significantly associated with metformin efficacy; the decrease in HbA1c and FBG was greater in GT + TT genotype than in GG genotype patients after metformin treatment (Figures 1 and 2). But the decrease in PBG in *STK11* rs2075604 genotypes was not significantly different. Apart from these, we found that the T allele in *STK11* rs2075604 had a better therapeutic metformin efficacy compared with the G allele in T2DM patients.

Although our study was rigorous and precise, several defects still existed. First, the difference of metformin efficacy may result from multiple genetic factors. Perhaps, other genes or SNPs will also influence metformin efficacy in these involved T2DM patients. Second, the phenotype is the result combined with the genotype and environment. Different living environments among different T2DM patients may also influence the progression of T2DM and influence the evaluation of metformin efficacy. Third, although the metformin dosage was not significantly different among patients in statistics, a different metformin dosage still exists among individuals due to security and medical reasons, which may affect the changes of HbA1c levels as well. Finally, the sample size should better be larger in our study, and we should validate the function of *STK11* rs2075604 in metformin efficacy into the a population.

In conclusion, the *STK11* rs2075604 polymorphism might have no impact on the incidence of T2DM among Chinese Han population but influences the therapeutic efficacy of metformin in treating Chinese T2DM patients. Our study gives evidence that the T allele of the *STK11* rs2075604 may be associated with better metformin efficacy. And the GT + TT genotype in *STK11* rs2075604 can decrease HbA1c level more significantly than the GG genotype after treatment of metformin. However, further pharmacogenetics and functional investigation should be conducted to examine the effect of *STK11* variations on metformin treatment to provide more powerful evidence for precise medicine.

## Figures and Tables

**Table 1:** Demographic and clinical characteristics of T2DM patients and healthy subjects.

Parameters	Healthy subjects (*n* = 200)	T2DM patients (*n* = 400)	*P*
Gender			
Male	111	245	0.177
Female	89	155	
Age (years)	56.88 ± 7.91	56.97 ± 12.65	0.925
BMI (kg/m^2^)	22.68 ± 1.69	24.46 ± 2.28	**0.000**
HbA1c (%)	5.53 ± 2.84	7.75 ± 1.75	**0.000**
FBG (mmol/L)	5.37 ± 1.55	7.82 ± 2.53	**0.000**
PBG (mmol/L)	6.56 ± 2.97	15.22 ± 6.43	**0.000**
INS (uIU/mL)	5.80 (3.69, 9.67)^∗^	7.99 (4.35, 12.70)^∗^	**0.000** ^∗^
CPO (ng/mL)	2.35 (1.81, 3.03)^∗^	1.89 (1.14, 2.75)^∗^	**0.000** ^∗^
PCPO (ng/mL)	7.48 ± 4.01	5.45 ± 3.44	**0.000**
TC (mmol/L)	5.17 ± 4.45	4.75 ± 1.27	0.082
TG (mmol/L)	1.68 ± 3.85	1.91 ± 1.88	0.340
LDL (mmol/L)	2.67 (2.15, 3.15)^∗^	2.81 (2.08, 3.38)^∗^	0.267^∗^
HDL (mmol/L)	1.40 (1.19, 1.68)^∗^	1.09 (0.93, 1.32)^∗^	**0.000** ^∗^

Data are shown as mean ± SD. Data was analyzed by two-sample *t*-test. ^∗^Skewed data shown as median (quartiles). Data was analyzed by the Mann–Whitney *U* test. BMI: body mass index; HbA1c: glycosylated haemoglobin; FBG: fasting blood glucose; PBG: postprandial blood glucose; INS: insulin; CPO: C-peptide; PCPO: postprandial C-peptide; TC: total cholesterol; TG: triglycerides; LDL: low-density lipoprotein cholesterol; HDL: high-density lipoprotein cholesterol; T2DM: type 2 diabetes mellitus. The numbers in bold indicated statistically significant values.

**Table 2:** Comparisons of allelic frequencies of *STK11* rs2075604 polymorphisms in T2DM patients and healthy subjects.

Genotypes	Healthy subjects (*n* = 200)	T2DM patients (*n* = 400)	*P*
*STK11* rs2075604			
GG	82 (41.0%)	164 (41.1%)	0.355
GT	87 (43.5%)	189 (47.4%)	
TT	31 (15.5%)	46 (11.5%)	
Alleles			
G	251 (62.7%)	517 (64.8%)	0.488
T	149 (37.2%)	281 (35.2%)	
HWE	0.326	0.445	

Data was analyzed by chi-square (*χ*^2^) test. T2DM: type 2 diabetes mellitus; HWE: Hardy-Weinberg equilibrium.

**Table 3:** Comparison of clinical characteristics between the responders and nonresponders before and after metformin treatment.

Parameters	Nonresponses (*n* = 32)	Responses (*n* = 62)	*P*
Gender			
Male	20	34	0.482
Female	12	28	
Age (years)	53.20 ± 10.50	52.61 ± 14.92	0.829
BMI (kg/m^2^)			
Baseline	24.27 ± 2.16	23.92 ± 1.74	0.391
After metformin	24.10 ± 4.02	23.32 ± 2.94	0.287
Mean change	−0.17 ± 4.39	−0.59 ± 2.96	0.581
Metformin dosage (mg/d)			
Baseline	1479.69 ± 278.78	1479.84 ± 171.172	0.997
Adjusted	1412.50 ± 235.89	1463.71 ± 375.61	0.484
Mean change	−67.19 ± 338.54	−16.13 ± 361.78	0.509
HbA1c (%)			
Baseline	6.33 ± 0.92	7.99 ± 1.57	**0.000**
After metformin	6.05 ± 0.69	5.18 ± 0.85	**0.000**
Mean change	−0.28 ± 0.74	−2.81 ± 1.57	**0.000**
FBG (mmol/L)			
Baseline	6.44 ± 2.83	8.35 ± 1.88	**0.000**
After metformin	5.80 ± 1.24	5.60 ± 1.10	0.462
Mean change	−0.83 ± 2.69	−2.83 ± 1.94	**0.000**
PBG (mmol/L)			
Baseline	10.84 ± 5.04	15.60 ± 10.79	**0.020**
After metformin	9.05 ± 1.85	8.99 ± 1.63	0.878
Mean change	−2.07 ± 5.11	−6.76 ± 10.98	**0.025**

Data was analyzed by two-sample *t*-test. BMI: body mass index; HbA1c: glycosylated haemoglobin; FBG: fasting blood glucose; PBG: postprandial blood glucose. The numbers in bold indicated statistically significant values.

**Table 4:** Association between *STK11* rs2075604 genetic polymorphism and metformin efficacy in T2DM patients (adjusted for age, gender, and BMI).

rs2075604 (G>T)	Non-response (*n* = 32)	Response (*n* = 62)	OR (95% CI)	*P*
Genotypes				
GG	18 (56.25%)	18 (29.03%)	1.0 (reference)	—
GT	11 (34.37%)	34 (54.84%)	3.091 (1.204–7.936)	**0.019**
TT	3 (9.38%)	10 (16.13%)	1.826 (0.886–3.762)	0.103
Alleles				
G	47 (73.44%)	70 (56.45%)	1.0 (reference)	—
T	17 (26.56%)	54 (43.55%)	2.133 (1.104–4.121)	**0.024**
GG versus GT/TT (dominant model)			3.143 (1.293–7.638)	**0.011**
GG/GT versus TT (recessive model)			1.721 (0.430–6.888)	0.443

Data was analyzed by the logistic regression by adjusting for sex, age, and BMI. OR: odds ratio; CI: confidence interval. The numbers in bold indicated statistically significant values.
